# Teaching nontechnical skills in the undergraduate education of health care professionals: a nationwide cross-sectional study in Hungary

**DOI:** 10.1186/s12909-024-05164-0

**Published:** 2024-02-22

**Authors:** Tamás Nagy, Gábor Fritúz, János Gál, Andrea Székely, Enikő Kovács

**Affiliations:** 1 Subdepartment of Clinical Simulation, Semmelweis University, P.O.B. 2, Budapest, H-1428 Hungary; 2 Faculty of Health Sciences, Semmelweis University, P.O.B. 2, Budapest, H-1428 Hungary; 3 Department of Anaesthesiology and Intensive Therapy, Semmelweis University, P.O.B. 2, Budapest, 1428 Hungary; 4 Heart and Vascular Center, Semmelweis University, P.O.B. 2, Budapest, H-1428 Hungary

**Keywords:** Nontechnical skills, Medical education, Nursing education, Cross-sectional study

## Abstract

**Background:**

The aim of our cross-sectional study was to evaluate the current situation and curriculum of nontechnical skills (NTS) training in the undergraduate education of health care professionals in Hungary.

**Methods:**

All institutes with relevant NTS training in Hungarian faculties of medicine and faculties of health sciences were asked to fill out a 19-item questionnaire. Descriptive statistics were performed, and the characteristics of NTS teaching and non-NTS teaching institutes were compared. The independent predictors of teaching NTS in a particular institute were identified with multiple logistic regression.

**Results:**

Seventy-seven institutes responded (52% response rate), of which 66% trained NTS. The most frequent method of NTS training is talking about them during a practice or lecture, and less than half of NTS respondents use simulation. The most frequent cause of not teaching NTS is a lack of human or technical resources. The type of faculty (*p* = *0.025*), academic year (*p* = *0.001*), field of medicine (*p* = *0.025*), and importance of teamwork (*p* = *0.021*) differed between NTS and noNTS institutes. Teaching students in academic year two represented the only independent predictor of NTS education (*p* = *0.012*).

**Conclusions:**

Our findings show that the undergraduate curriculum of Hungarian universities includes some type of NTS education; however, this education requires further development.

**Supplementary Information:**

The online version contains supplementary material available at 10.1186/s12909-024-05164-0.

## Background

Nontechnical skills (NTS), originating from aviation and high-risk industries, play a crucial role in modern medicine to secure patient safety and high-quality patient management not only in emergency medicine, anaesthesiology, or surgery but also in all fields of medicine [1]. NTS can be defined as “a constellation of cognitive and social skills, exhibited by individuals and teams, needed to reduce error, and improve human performance in complex systems” [2]. Including but not limited to the most important NTS applied in health care are communication, situational awareness, decision-making, prioritization, leadership, teamwork, and the ability to process and incorporate feedback [3]. The UK General Medical Council already incorporated these skills as key elements in the Council’s Generic Professional Capabilities Framework [4]. Furthermore, NTS are increasingly prominent skills required in modern medicine and health care to secure high-quality and first safe patient management [1, 3, 5–7].

Even though NTS education is gaining prominence worldwide, it also needs to be highlighted that the methods and types of NTS education are very heterogeneous among nations, fields of medicine, and levels of health care professionals, resulting in the requirement of further investigation to identify the factors that can help improve the development of proper curriculum in NTS training and not least patient management [1, 8, 9].

The aim of our study was to investigate the current situation of NTS teaching in undergraduate health care education in Hungary. No previous research has examined the circumstances of Hungarian NTS training, and the current study serves as a first step in developing a national curriculum targeting undergraduate NTS education.

## Methods

### Study design

We performed a prospective study based on a nationwide cross-sectional survey conducted in Hungary between 1 December 2021 and 1 December 2022 to assess the current situation of teaching NTS in the undergraduate education of health care professionals. The online, anonymous questionnaire was sent to all Hungarian universities and institutes, departments and clinics that take part in the education of health care professionals providing bachelor’s, master’s, or doctoral degrees, and NTS are relevant in the specialties taught by the institutes. To reach a satisfactory response rate, emails were sent to the heads of the institutes three times during the study period. In addition, all the institutes received a phone call as a reminder to complete the questionnaire. We expected one completed questionnaire from all participating institutes.

The Semmelweis University Regional and Institutional Committee of Science and Research Ethics approved our study (approval number: 161/2021). The participants were informed that by filling out the questionnaire, they would provide informed consent for anonymized data analysis and potential data presentation.

### Participants

We included in our study all Hungarian universities that teach undergraduate health care education. The universities were chosen based on the Hungarian Educational Authority database. There are four universities in Hungary providing Faculty of Medicine (Table 1). In addition, all these universities offer Faculty of Health Sciences with different institutes and departments educating various health care specialties (e.g., nursing, paramedics, midwives, health visitors, etc.). Moreover, there are four additional universities providing some education in the Faculties of Health Sciences (Table 1).

**Table 1:** List of Hungarian universities that take part in undergraduate health care education

**University**	**Faculty of Medicine**	**Faculty of Health Sciences**
Semmelweis University	yes	yes
University of Pécs	yes	yes
University of Szeged	yes	yes
University of Debrecen	yes	yes
University of Győr	no	yes
Gál Ferenc University	no	yes
University of Miskolc	no	yes

After selecting the appropriate universities, the list of all institutes belonging to a particular university was screened by two authors (TN, EK). The screened institutes belonged to the faculties of medicine or health sciences; however, some of them provided courses on more faculties. We excluded institutes that teach subjects and specialties with no relevance regarding NTS (e.g., institutes teaching anatomy, biochemistry, biology, biophysics, pathology, laboratory medicine, etc.). The list of included institutes can be found in Additional file 1. Finally, 148 Hungarian institutes were included in the study, and questionnaires were sent to the heads of the included institutes, departments, and clinics. The head of the institute was asked to nominate one person to fill out the questionnaire who was familiar with the structure and content of the institute’s curriculum.

### Questionnaire

We developed a 19-item online questionnaire with 17 multiple choice questions, one Likert-scale question and one open-ended question (Additional file 2) in Google Forms® (Google Inc.). The multiple-choice questions covered demographic data and questions about the actual quality, condition, and implementation of NTS education in a particular institute. We also investigated the causes if an institute did not train NTS. A six-point Likert-scale question was used to measure whether there is a requirement to develop NTS education in institutes that already teach NTS based on the respondents’ opinion (answers: 1 - strongly disagree, 2 - disagree, 3 - somewhat disagree, 4 – somewhat agree, 5 – agree, 6 - strongly agree). The open-ended question asked participants to list all subjects taught by one institute. The first page of the questionnaire described the purpose and design of the study. Only after agreeing to participate and potential publication of data could respondents continue with the responses.

A complex (internal and external) questionnaire validation process was performed based on the Association for Medical Education recommendations [10]. As a first step after the development of the questionnaire (TN), two investigators (EK, ASz) reviewed it to assess content and intelligibility. After the internal validation part, four experts in the field of NTS education from the Semmelweis University Faculty of Medicine and Faculty of Health Sciences evaluated the questionnaire. After the evaluation, the suggested changes were made in the content or structure of the questionnaire. This process was followed by a written cognitive interview with six educators from Hungarian universities. The steps of questionnaire development can be seen in Additional file 1.

### Statistical analysis

We used descriptive statistics to describe the characteristics of the respondents and to provide an overview of the current practice of teaching NTS in Hungary. Both the answers given to multiple choice questions and the open-ended question were categorized, and frequencies with percentages were determined. Median and interquartile ranges were calculated from the answers given to the Likert-scale question.

Less than 5% of the data were missing due to some incompletely answered questions. Missing data were excluded from the analysis.

We compared the characteristics of NTS teaching institutes (NTS institutes) with the characteristics of non-NTS teaching institutes (noNTS institutes) in the relevant variables. The chi-square test or Fisher’s exact test (if the frequency of a category was less than 5) was used for comparison. The level of significance was set at *p* < 0.05.

In addition, multiple logistic regression was performed to determine which factors influence whether an institute trains NTS in undergraduate education. Teaching NTS or not was recorded as the dependent variable. Independent variables were chosen based on the results of the univariate analysis: if there was a significant difference in a variable between the NTS and noNTS groups, the variable was included in the multiple logistic regression. Odds ratios and levels of significance were determined. The level of significance was set at *p* < 0.05.

Statistical analysis and figures were performed using GraphPad Prism version 9.3.1 (GraphPad software, La Jolla, CA).

## Results

A total of 77 institutes participated in our survey, resulting in a response rate of 52%. The confidence interval of the response rate fits at 95% with a margin of error of 8%.

### Characteristics of participants

**Fig. 1:**
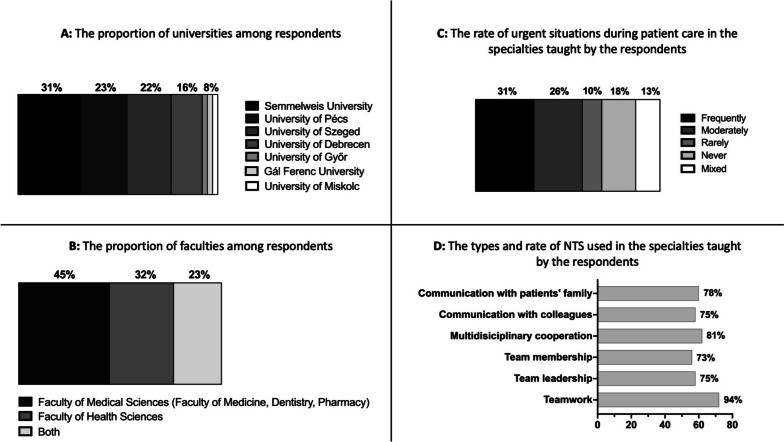
The results of descriptive statistics characterizing participants. Number of respondents: *N* = 77, response rate 52%. **A** The proportion of Hungarian universities among the respondents. **B** The proportion of faculties among the respondents. The category “Faculty of Medical Sciences” reflects respondents teaching in faculties providing doctoral degrees at the end of their undergraduate education (medical doctors, dentists and pharmacists). The category “Faculty of Health Sciences” reflects respondents teaching in faculties that educate health care workers. Category “Both” reflects faculties that educate students in both previous categories. **C** The rate of urgent situations during patient care in the subject and specialty taught by a given responder. **D** The types and rate of nontechnical skills used in the specialties taught by the respondents. NTS: nontechnical skills

Figure 1 and Table 2 show the characteristics of the participating institutes. We received answers from seven universities, which represent all faculties that teach various aspects of the health care profession in Hungary. All four Hungarian medical universities (Semmelweis University, University of Pécs, University of Szeged and University of Debrecen) participated in the survey (Fig. 1A). Despite multiple attempts, we did not receive any responses from a single small university (Károli Gáspár University of the Reformed Church in Hungary) that offers nursing education. Fourty-five percent of the respondents teach in faculties of medical sciences providing a doctoral degree (Medical Doctor, Doctor of Dentistry, Doctor of Pharmacy), 32% of the respondents teach in faculties of health sciences (educating nurses, paramedics, etc.) and 23% of respondents take part in education of both types of faculties (Fig. 1B). One-third of the participating institutes teach specialties, in which the rate of emergency situations is high based on the respondents’ opinion, and 14% of them answered that there are no urgent situations in their taught specialty (Fig. 1C). Almost all respondents (94%) stated that teamwork has been important in their educated field. Moreover, multidisciplinary cooperation and communication with patients, relatives or colleagues were the most frequent aspects of NTS characterizing the participating institutes’ specialties (Fig. 1D).

**Table 2:** Comparison of groups teaching nontechnical skills (NTS group) and not teaching nontechnical skills (noNTS group)

	**Responses total**	**Teaching NTS**	**Not teaching NTS**	***P***
	n (%)	n (%)	n (%)	
Total	77 (100%)	51 (66%)	26 (34%)	
*University*				
Budapest	24 (31%)	15 (29%)	9 (35%)	0.56*
Pécs	18 (23%)	11 (22%)	7 (27%)	
Szeged	17 (22%)	12 (24%)	5 (19%)	
Debrecen	12 (16%)	7 (14%)	5 (19%)	
Other	6 (8%)	6 (11%)	0	
*Faculty*				
Faculty of medical sciences	34 (44%)	17 (34%)	17 (65%)	**0.025**
Faculty of health sciences	24 (31%)	20 (40%)	4 (16%)	
Both	18 (23%)	13 (26%)	5 (19%)	
*Academic year*				
Teaching students in year 1	30 (39%)	24 (47%)	6 (23%)	0.051
Teaching students in year 2	29 (38%)	26 (51%)	3 (12%)	**0.001**
Teaching students in year 3	36 (47%)	28 (55%)	8 (31%)	0.090
Teaching students in year 4	46 (60%)	32 (64%)	14 (54%)	0.472
Teaching students in year 5	29 (38%)	16 (31%)	13 (50%)	0.111
Teaching students in year 6	23 (30%)	12 (24%)	11 (42%)	0.090
*Specialty*^*a*^				
Emergency medicine, anaesthesiology or intensive therapy	9 (12%)	7 (14%)	2 (8%)	**0.025**
Specialties with surgical or interventional aspects	17 (22%)	5 (10%)	12 (46%)	
Specialties with conservative treatment methods	29 (38%)	22 (43%)	9 (35%)	
Specialties focusing on health system development	20 (26%)	17 (33%)	3 (12%)	
*The rate of acute situations in the taught specialty*				
Frequent	24 (31%)	17 (33%)	7 (28%)	0.545
Moderate	20 (26%)	11 (22%)	9 (36%)	
Rare	8 (10%)	7 (14%)	1 (4%)	
Extremely rare	14 (18%)	9 (18%)	5 (20%)	
Mixed^b^	10 (13%)	7 (14%)	3 (12%)	
*Types of NTS characterizing the taught specialty*				
Teamwork	72 (94%)	48 (94%)	24 (92%)	0.552
Team leadership	57 (74%)	41 (80%)	16 (62%)	0.067
Team membership	57 (74%)	42 (82%)	15 (60%)	**0.021**
Multidisciplinary cooperation	61 (79%)	41 (80%)	20 (77%)	0.469
Communication with colleagues	57 (74%)	40 (78%)	17 (65%)	0.168
Communication with family	61 (79%)	42 (82%)	19 (73%)	0.254
*The willingness of participating in a course about NTS*				
The rate of institutes requiring a course about NTS education	69 (90%)	46 (90%)	23 (88%)	0.685

The chi-square test or Fisher’s exact test was performed, and the level of significance was set at *p* < 0.05

*NTS* Nontechnical skills, *n* Number of respondents

^*^Category “other” was waived from the statistical calculation, as the value in the noNTS group was 0

^a^The answers regarding the characteristics of a particular specialty taught by the respondents

^b^Mixed: the respondents teach more subjects, which include specialties with different ratios of emergency situations

Most participants teach students in several academic years: most frequently in academic year four and least frequently in academic year six (Table 2). Thirty-eight percent of the respondents teach a field that is characterized by conservative treatment methods, 26% teach specialties focusing on health system organization, 22% teach surgical or interventional courses and 12% teach emergency medicine, anaesthesiology, or intensive therapy (Table 2).

**Fig. 2:**
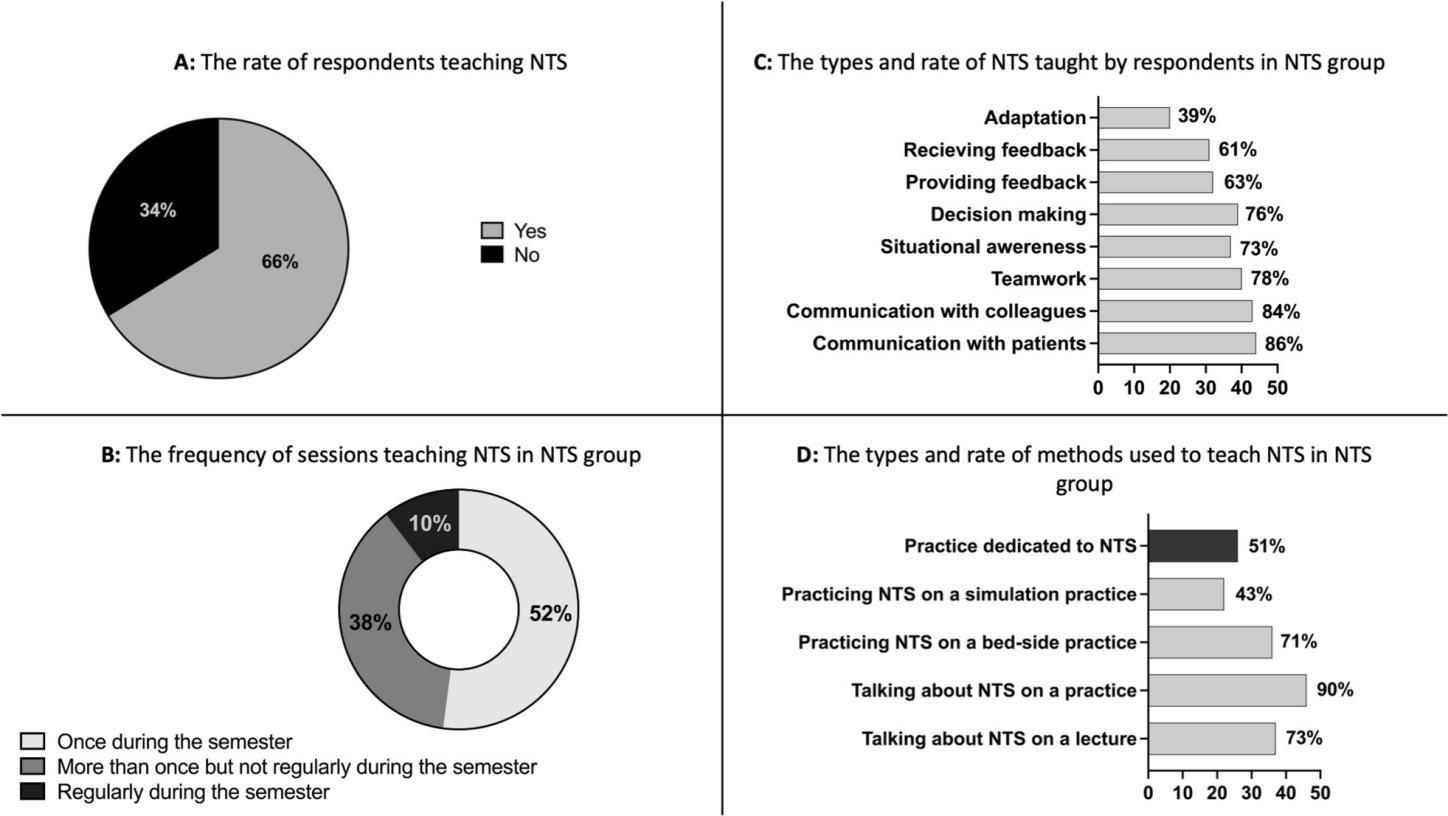
Characteristics of participating institutes teaching NTS (*n* = 51). **A** The rate of respondents/institutes teaching NTS. Yes: the institute teaches NTS in undergraduate education. No: the institute does not teach NTS in undergraduate education. **B** The frequency of sessions teaching nontechnical skills in the NTS group. **C** The types and rates of nontechnical skills taught by respondents in the NTS group. X axis: number of responses. **D** The types and rates of methods used to teach nontechnical skills in the NTS group. X axis: number of responses. NTS: nontechnical skills

**Fig. 3:**
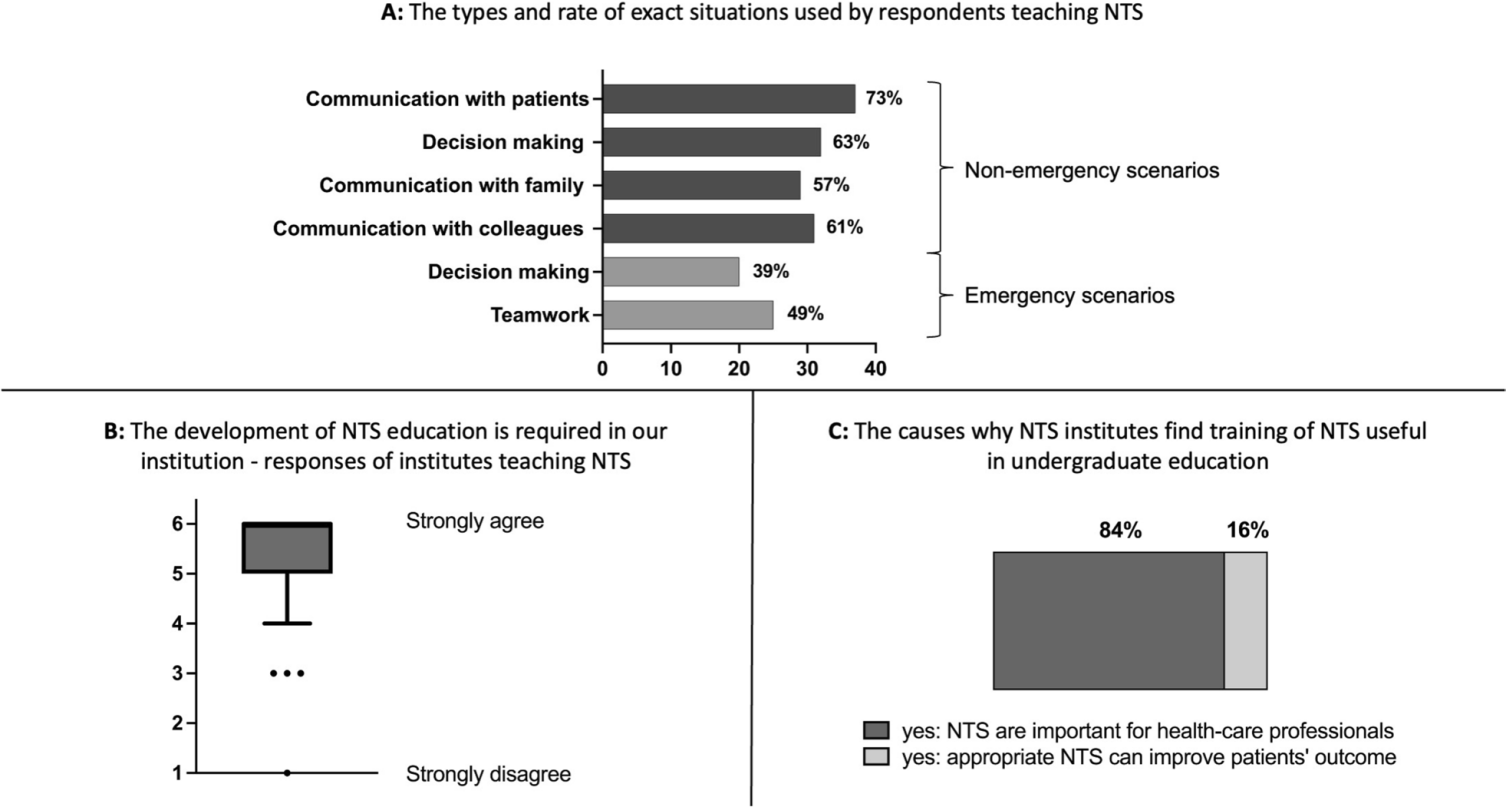
Characteristics of NTS education in the NTS group (*n* = 51). **A** The types and rate of exact situations used by respondents to train NTS. X axis: number of responses. **B** The median value and interquartile ranges of Likert-scale answers given to question if NTS education requires further development in a particular institute. Answers: 1 - strongly disagree, 2 - disagree, 3 - somewhat disagree, 4 – somewhat agree, 5 – agree, 6 - strongly agree. Box and whisker plots were generated by the Tukey method. **C** The rate of reasons why an institute finds training NTS important in undergraduate education. NTS: nontechnical skills

Based on the answers, 51 (66%) participating institutes train NTS in undergraduate education (Fig. 2A). Fifty-two percent of these 51 institutes focus on NTS only once in a semester, 38% deal with NTS more than once, and only 10% teach NTS on a regular basis during their courses (Fig. 2B). Figure 2C summarizes the types of NTS that are taught by respondents who teach NTS in undergraduate education (NTS institutes). Figure 2D shows the methods used to transfer NTS. The most frequent method is talking about NTS in a lecture or in practice, and only 51% of respondents dedicate a whole session to NTS. However, 71% of institutes incorporate practising NTS into bed-side trainings. Moreover, Fig. 3A summarizes the exact scenarios and situations applied by the NTS teaching institutes.

Most institutes actively training NTS strongly agree that there is a requirement to develop their NTS educational methods (median value of answers given to Likert-scale question: 6; IQR: 5, 6) (Fig. 3B). Eighty-four percent of these institutes teach NTS because these skills are important for health care professionals, and 16% train NTS due to their influence on patients’ outcomes. None of the NTS institutes teach NTS only because this is the trend today, and no institute thinks that teaching NTS is not important (Fig. 3C).

**Fig. 4:**
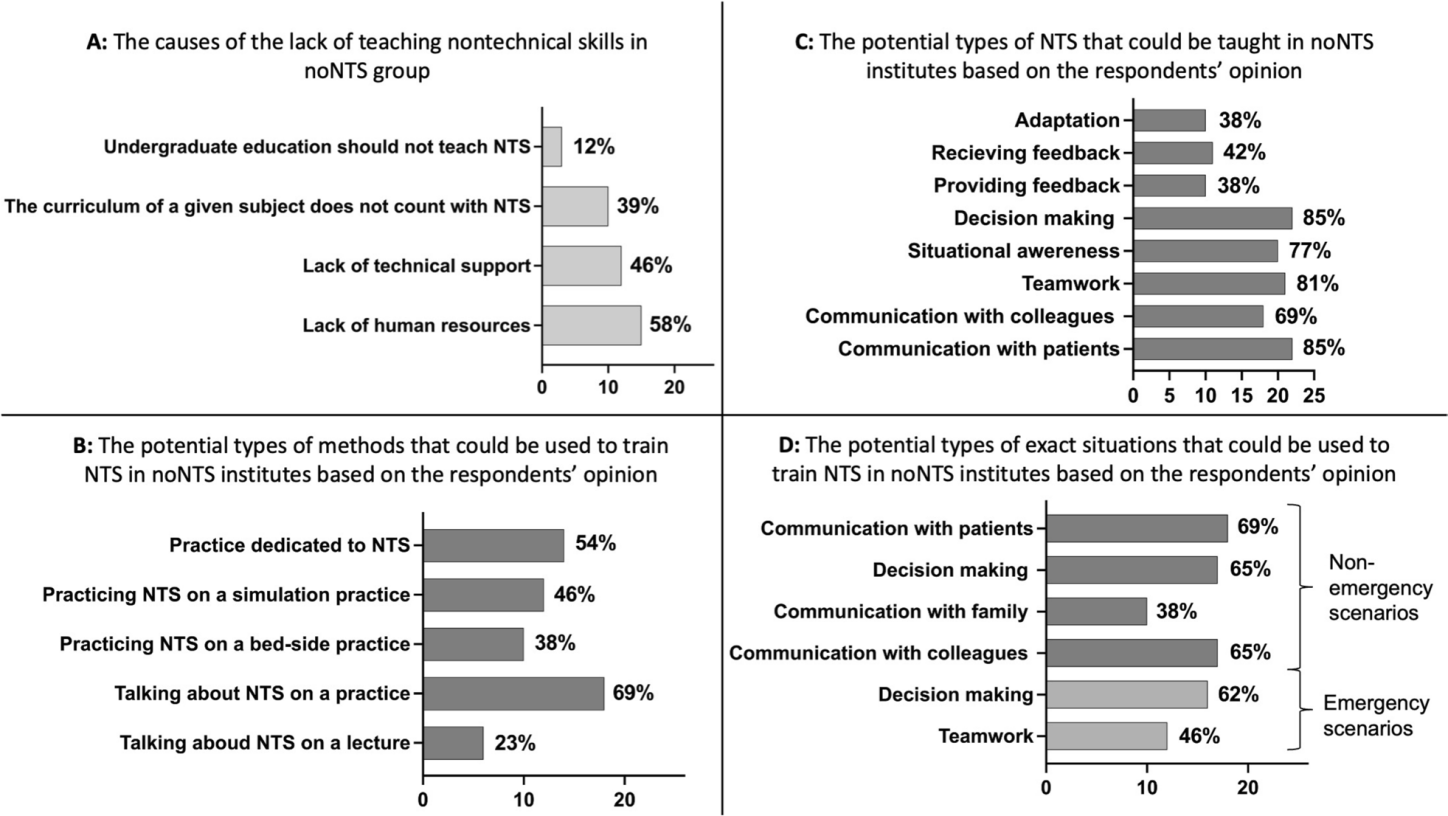
Characteristics of participating institutes not teaching NTS (*n* = 26). **A** The causes of the lack of teaching nontechnical skills in the noNTS group. **B** The potential types of methods that could be applied to train NTS in institutes not teaching NTS in undergraduate education based on the respondents’ opinions. X axis: number of responses. **C** The potential types of NTS that could be taught in noNTS institutes based on the respondents’ opinions. X axis: number of responses. **D** The potential types of exact situations that could be used to train NTS in noNTS institutes based on the respondents’ opinions. X axis: number of responses. NTS: nontechnical skills

Twenty-six (34%) participating institutes do not teach NTS in undergraduate education (noNTS institutes). The most frequent causes of not teaching NTS are the lack of human or technical resources; however, 12% of these institutes think that these skills should not be taught during undergraduate education (Fig. 4A). Figure 4B-D present the potential types, methods and exact teaching situations that can be taught and applied by the noNTS institutes in the future.

Only 50% of institutes not teaching NTS are planning to introduce the education of these skills into their curriculum in the future.

### Factors influencing NTS education

No differences were found between NTS institutes and noNTS institutes regarding the following factors: the university to which they belong, the rate of urgent situations in the taught specialty, the rate and type of most NTS characterizing the field, and the willingness to participate in a methodological course about NTS (Table 2). The proportion of faculty types showed a significant difference between NTS and noNTS institutes (*p* = *0.025*). More noNTS institutes belonged to faculties of medical sciences, and more NTS institutes belonged to faculties of health sciences. Moreover, significantly more NTS institutes educated students in academic year two (*p* = *0.001*). However, there was no difference between NTS institutes and noNTS institutes in other academic years. In addition, we found a significant difference between the two groups in the type of specialty taught (*p* = *0.025*). The proportion of surgical specialties was higher among noNTS institutes, and the proportion of specialties of health system development was higher among NTS institutes. Furthermore, more participants judged in the NTS group that the ability to be a team member is important in their field (*p* = *0.021*).

Finally, the results of multiple logistic regression (Table 3) show that the academic year and teaching students in academic year two represent independent predictors of NTS education (*p* = *0.012*). The type of faculty, specialty, and the importance of being a team member in a particular field are not independent predictors of teaching NTS based on our analysis. However, specialties other than surgical or interventional fields and the importance of team membership showed a tendency to predict whether an institute teaches NTS in Hungary.

**Table 3:** Factors influencing whether an institution educates nontechnical skills or not in undergraduate medical education in Hungary

	**Odds ratio (95% CI)**	***P***
*Faculty*		
Faculty of medical sciences	Reference	
Faculty of health sciences	0.19 (0.01–2.24)	0.234
Faculty of medical and health sciences	1.22 (0.25–6.05)	0.801
*Academic year*		
Not teaching in academic year 2	Reference	
Teaching in academic year 2	23.53 (1.84–565.3)	**0.012**
*Specialty*		
Emergency medicine, anaesthesiology and ICU	6.30 (0.88–63.63)	0.082
Specialties with surgical or interventional aspects	Reference	
Specialties with conservative treatment methods	4.40 (1.03–21.61)	0.054
Specialties focusing on health system development	8.17 (1.04–97.18)	0.062
*Types of NTS characterizing the taught specialty*		
Team membership is NOT important	Reference	
Team membership is important	3.65 (1.01–14.67)	0.055

Multiple logistic regression was performed

The level of significance was set at *p* < 0.05

*CI* Confidence interval

## Discussion

This is the first study examining NTS education in Hungary. The objective of our cross-sectional questionnaire-based study was to explore the current situation of teaching NTS in Hungarian undergraduate health care and medical education and to investigate the factors influencing the willingness, feasibility, and potential barriers to NTS education in universities in Hungary.

We studied all Hungarian universities providing some type of health care education, as proper NTS is important not only for medical doctors but also for nurses and all professionals involved in the health care system [2, 11]. The institutions participating in our survey find NTS education important; however, only two-thirds of them incorporate NTS teaching into their curriculum. Moreover, regular sessions educating NTS were found only in 10% of the NTS group. The literature shows a variability in the incidence of NTS teaching among countries especially in the undergraduate level, and not only the Hungarian situation is so poor. A survey held in 2006 in the US and Canada found that only 25% of included institutions reported a curriculum incorporating patient safety issues and NTS teaching [12]. A novel study investigating the prevalence and content of NTS in undergraduate education in anaesthesiology and surgery in Canada found that NTS teaching is poorly introduced in undergraduate perioperative education and that improvement is needed [13]. One of the reasons of underrepresenting NTS teaching in the undergraduate education may be the lack of universal NTS training framework for undergraduate students. There is increasing evidence of the importance of proper application of NTS in medicine to ensure safe and high-quality patient management [14–16]. However, most of data are from emergency medicine, anaesthesiology, intensive therapy, and surgical fields. In addition, there are growing requirements for learning NTS and high-quality NTS education not only at the postgraduate level but also at the undergraduate level [17–19]. The World Health Organization developed a curriculum on patient safety for medical students facilitating education on patient safety management focusing on team-based training and error prevention [20]. The American Medical Association also aimed to enhance the curriculum of undergraduate medical students and included NTS education as a part of patient safety topics [21]. The principle of introducing NTS into the undergraduate curriculum is to deliver this knowledge to future health care professionals before their professional attitude is fully developed [8]. A recent systematic review identified 68 articles investigating the circumstances of NTS teaching in the undergraduate education of health care professionals and created a proposal to coordinate the future development of NTS training, highlighting that all countries and universities should create a curriculum on NTS teaching in medical and healthcare education for undergraduate students [22]. There is a huge requirement to enhance the curriculum of universities with high-quality NTS education not only because of its impact on patient safety but also because it supports students’ self-confidence and performance and reduces anxiety when interacting with patients [23].

We also investigated the methods used during NTS education. We found that the most frequent ways to teach NTS were talking about them during a practice or lecture or even incorporating some NTS tasks into bed-side training. Simulation as a method to teach NTS is used in less than half of NTS educating institutes based on the answers of our respondents. However, the current literature finds simulation to be one of the best techniques for NTS teaching [18, 24]. Nicolaides et al. also suggest applying some type of simulation training to improve NTS among undergraduate students [22]. The small number of simulation trainings in Hungary can be explained by the high costs and human resource requirements of simulation sessions. In addition, this method is widely spread in the fields of anaesthesiology, intensive therapy, and emergency medicine; however, we investigated all fields of medicine and health care in which some type of NTS is needed. Moreover, not only a national curriculum is needed on NTS teaching but a universal training on the methodology of NTS education.

There are ample NTS that are required to secure safe patient management, which can be categorized into more groups: performance shaping factors; planning, preparation, and prioritization; situation awareness; decision-making; communication; teamwork; and leadership [2]. The most frequently educated NTS in Hungarian undergraduate health care education are communication, teamwork, situational awareness, and decision-making. This coincides with other findings in the current literature showing that communication skills and teamwork are the most frequent NTS trained in undergraduate education [22]. These types of NTS are common and can be taught with simpler methods requiring less preparation and training of instructors.

A third of our respondents do not teach NTS in undergraduate education at all. The most frequent explanations were the lack of human and technical resources, and only 12% of respondents in the noNTS group answered that NTS should not be taught in undergraduate education. The education of NTS is indeed a time-consuming process requiring special instructor training and preparation. Moreover, the most effective ways of teaching NTS are simulation training with feedback or debriefing requiring additional human resources [22, 24]. A solution to these deficiencies would be the introduction of a national framework for NTS education with a clear guidance on methodology, and suggestions for the economical use of human and technical resources.

We also aimed to explore the factors that can influence the incidence of NTS teaching. We found that institutes belonging to faculties of health sciences incorporated NTS training in their curriculum more frequently than faculties of medical sciences. This can be explained by the fact that faculties of health sciences lead a more practical way of teaching with less theory, in contrast to faculties of medical sciences, where transferring theoretical knowledge has a higher priority. However, it needs to be highlighted that the type of faculty was not an independent predictor of educating NTS in a Hungarian institute. It is also an important finding that the institutes educating specialties with surgical or interventional methods were less involved in NTS training than other specialties. However, the current literature data indicate that NTS is important in surgical fields to secure patient safety, and these fields should include NTS training in their curriculum [5, 17, 18].

Institutes educating students in the lower academic years were more involved in NTS training than institutes educating students in the higher academic years. In addition, this was an independent predictor of teaching NTS in Hungarian universities. This is an interesting finding, as more clinical subjects are taught in the higher academic years. We are planning to investigate the cause of this in more detail in the future.

Based on our findings, we can conclude that Hungarian universities educating health care professionals include some type of NTS training in undergraduate education. However, the frequency and methods of NTS education vary among the institutes educating at these universities, and the condition of NTS training requires further improvement in Hungary. Almost all respondents agree and are open to involvement in the development of NTS education. We are planning to develop methods, courses, and further investigations to improve the current situation of NTS training in Hungary.

### Limitations of the study

Our study has some limitations that need to be considered. The response rate was only 52%; however, the confidence interval and margin of error were acceptable. We included not only some special fields in medicine but also all disciplines where NTS is used in some way, resulting in a very heterogeneous population of respondents. The questionnaire was evaluated in more steps and contains complex questions; however, there may be some aspects that were not analysed regarding NTS education in our country. Moreover, the complexity of the questions and the reliance on self-reporting also could introduce some biases.

Despite these limitations, our study is the first investigation exploring NTS teaching in Hungarian undergraduate health care education and provides the basis for the development of the undergraduate curriculum and future research in the topic.

### Supplementary Information

**Supplementary Material 1.**

**Supplementary Material 2.**

## Data Availability

The datasets supporting the conclusions of this article can be requested from the author Enikő Kovács (kovacs.eniko2@med.semmelweis-univ.hu).

## Abbreviation

NTS: Nontechnical skills
