# Supplementary material for: Teaching nontechnical skills in the undergraduate education of health care professionals: a nationwide cross-sectional study in Hungary
Source: BMC Med Educ. 2024 Feb 22;24:174. doi: 10.1186/s12909-024-05164-0 (PMC10885394; doi:10.1186/s12909-024-05164-0)
Supplement: Supplementary file 1 — Supplementary Material 1. [file 12909_2024_5164_MOESM1_ESM.pdf]

Table 1. The list of institutes included in our study and received the questionnaire

University	Faculty	Institute (Department, Institute, Clinic)
University of Pecs	Medical School	Institute of Behavior - Department of Clinical Studies
		Department of Operational Medicine
		Department of Family Medical
		Department of Forensic Medicine
		Medical Public Health Institute
		Surgery Institute and Research Institute
		Translational institute
		Department of Anesthesiological and Intensive Therapy
		Department of Dermatology, Venereology and Oncodermatology
		Department of Vascular Surgery
		Department of Emergency Medicine
		Dental and Oral Surgery Clinic
		Department of Othorinolaryngology
		Department of Pediatrics
		1st Department of Medicine
		Department of Neurosurgery
		2nd Department of Internal Medicine and Nephrological Center
		Department of Neurology
		Department of Oncotherapy
		Department of Orthopaedics
		Department of Medical Rehabilitation and Physical Medicine
		Department of Psychiatry and Psychotherapy
		Department of Surgery
		Department of Ophtalmology
		Heart Institute

University of Szeged	Faculty of Health Sciences	Department of Obstetrics and Gynecology<br>Departmant of Traumatology and Hand Surgery<br>Department of Urology<br>Institute of Basics of Health Sciences, Midwefery and Health Visiting<br>Institute of Health Insurance<br>Institute of Physiotherapy and Sport Science<br>Institute of Emergency Care, Pedagogy of Health and Nursing Sciences<br>Institute of Diagnostics<br>Institute of Nutritional Science and Dietetics
	Albert Szent-Györgyi Clinical Center	Department of Anaesthesiology and Intensive Therapy<br>Department of Oral and Maxillofacial Surgery<br>First Department of Internal Medicine<br>Second Department of Internal Medicine and Cardiological Center<br>Department of Dermatology and Allergology<br>Family Medicine Department<br>Department of Oto-Rhino-Laryngology and Head- Neck Surgery<br>Department of Pediatrics and Pediatric Health Care Center<br>Department of Neurosurgery<br>Department of Neurology<br>Department of Oncotherapy<br>Department of Orthopaedics<br>Department of Psychiatry<br>Department of Air and Space Medicine<br>Department of Surgery<br>Department of Ophtalmology<br>Department of Obstetrics and Gynecology<br>Department of traumatology

		Department of Pulmonology
		Department of Urology
	Albert Szent-Györgyi Faculty of Medicine	Institute of Health Economics
		Institute of Behaviour
		Institute of Public Health
		Department of Preventive Medicine
		Rehabilitation Medicine Department
		Clinical Skills Centre
		Department of Sports Medicine
	Faculty of Health Sciences and Social Studies	Department of Nursing
		Department of Health Sciences and Health Management
		Section of Health Behaviour and Health Promotion
		Department of Physiotherapy
		Preventive Health Care Department
		Department of Social Work and Social Policy
		Department of Family Medicine and Occupational Health
University of Debrecen	Clinical Center	Institute of Behaviour
		Department of Family Medicine and Occupational Health
		Institute of Public Health and Epidemiology
		Department of Anesthesiology and Intensive Therapy
		Department of Internal Medicine
		Department of Dermatology
		Othorinolaryngology and Head and Neck Surgery Clinic
		Department of Orthopaedics
		Department of Medical Rehabilitation and Physical Medicine
		Pediatrics Clinic

		Clinic of Cardiology
		Neurological Clinic
		Surgical Clinic
		Clinic of Ophthalmology
		Obstetrical Clinic
		Urological Clinic
	Faculty os Health Sciences	Department of Nursing and Midwifery
		Department of Theoretical and Integrative Health Sceinces
		Department of Emergency and Oxyology
		Department of Social Sciences
		Department of Methodology and Prevention
	Faculty of Public Health	Department of Physical Therapy
		Department of Health Promotion
		Department of Health Management and Quality Management
		Department of Habilitation Medicine
		Department of Interventional Epidemiology
**University of Győr**	Faculty of Health and Sports Science	Department of Health Sciences
**Semmelweis University**	Faculty of Medicine	Department of Family Medical
		Department of Neurosurgery
		Institute of Public Health
		Department of Oncology
		Department of Rehabilitational Medicine
		Department of Rheumatology and Clinical Immunology
		Department of Traumatology
		Subdepartment of Neurotraumatology
		Subdepartment of Sports Surgery

	Faculty of Health Sciences	Department of Anaesthesiology and Intensive Therapy<br>Department of Internal Medicine and Hematology<br>Department of Endocrinology<br>Department of Dermatology, Venereology and Oncodermatology<br>Department of Otorhinolaryngology, Head and Neck Surgery<br>1st Department of Paediatrics<br>2nd Department of Paediatrics<br>Department of Clinical Psychology<br>Institute of Behavioural Sciences<br>Department of Breast Surgery at the base of the National Institute of Oncology<br>Department of Neurology<br>Department of Orthopaedics<br>Department pf Psychiatry and Psychotherapy<br>Department of Pulmonology<br>Department of Surgery and Interventional Gastroenterology<br>Department of Emergency Medicine<br>Department of Ophtalmology<br>Heart and Vascular Center<br>Department of Addictology<br>Department of Psychology<br>Department of Nursing Studies<br>Department of Family Methodology<br>Department of Dietary and Nutrition<br>Department of Health Sciences<br>Department of Physiotherapy<br>Department of Public Health<br>Department of Oxiology and Emergency Care

		Department of Obstetrics and Gynecology
		Department of Social Sciences
	Faculty of Public Services	Digital Institute of Health Sciences
		Institute of Mental Hygiene
	Faculty of Andras Pető	Institute of Human Sciences
		Conductive Pedagogy Institute
**Gál Ferenc University**	Faculty of Health and Social Science	Department of Nursing Science
		Institute of Health and Social Science
		Department of Health Tourism
		Department of Education and Social Psychology
		Department of Social Pedagogy
**Károli Gáspár University**	Faculty of Social and Health Sciences	Institute of Health Sciences
		Social Work and Diacona Institute
		Department of Social Work and Social Studies
**University of Miskolc**	Faculty of Health Sciences	Theoretical Institute of Health Sciences
		Theoretical Department of Health Sciences and Health Organizing
		Department of Physiotherapy

Table 2. The steps of developing and distributing the questionnaire.

**Step 1**	Questionnaire development
**Step 2**	Review by two investigators to assess if items were clear and written in accordance with current best practices in questionnaire design.
**Step 3**	Four experts in the field of NTS education evaluated content, intelligibility of items and relevance.
**Step 4**	Changes were performed based on the recommendations of the experts involved in step 3.
**Step 5**	Six educators participating in Hungarian undergraduate education of health-care professionals went through a written cognitive interview to ensure responders' proper understanding and interpretation.
**Step 6**	The recommended changes were incorporated in the questionnaire.
**Step 7**	Questionnaire sent to potential responders, who received three reminders in email and one phone call during the study period.
