# Supplementary material for: Teaching nontechnical skills in the undergraduate education of health care professionals: a nationwide cross-sectional study in Hungary
Source: BMC Med Educ. 2024 Feb 22;24:174. doi: 10.1186/s12909-024-05164-0 (PMC10885394; doi:10.1186/s12909-024-05164-0)
Supplement: Supplementary file 2 — Supplementary Material 2. [file 12909_2024_5164_MOESM2_ESM.pdf]

Questionnaire about the situation of NTS teaching in Hungarian undergraduate healthcare education

	Question	Answer options	Answer type
1.	Which university do you belong to?	Semmelweis University<br>University of Pécs<br>University of Szeged<br>University of Debrecen<br>University of Győr<br>Gál Ferenc University<br>University of Miskolc	MCQ
2.	In which faculties does your institute teach in the undergraduate education? More options are available.	Faculty of Medicine<br>Faculty of Health Sciences<br>Faculty of Dentistry<br>Other, please specify	MCQ
3.	Which subjects/specialties do you teach during undergraduate education?		Open ended
4.	In which academic years does your institute educate during the undergraduate education? More options are available.	1<br>2<br>3<br>4<br>5<br>6	MCQ

5.	Which of the followings charaterize the specialty you teach regarding the urgency of the situations in the given specialty?	Emergency situations occur regularly	MCQ
		Emergency situation occur moderately	
		Emergency situation occur rarely	
		There are no emergency situations in our specialty	
		We teach more subjects/specialties with different rate of urgent situations	
6.	Which of the followings characterize non-technical skills in the particular specialty you teach in undergraduate education? You can choose more answers.	Team work is important.	MCQ
		Our professionals often lead teams.	
		Team membership is important in our field.	
		Multidisciplinary cooperation is improtant in our field.	
		Our professionals often need to communicate with colleagues.	
		Our professionals often need to communicate with patients and relatives.	
		Other, please specify	
7.	Does yout institute consciously teach non-technical skills (NTS) during undergraduate education?	yes	MCQ
		no	

8.	Would you participate in methodological course about how to educate NTS?	yes<br>no	MCQ
	Next questions should be applied if you teach NTS.		
9.	Which NTS does your institute teach during undergraduate education? You can choose more answers.	Communication with patients<br>Communication with colleagues<br>Teamwork<br>Situational awerness<br>Decision making<br>Adaptation<br>Receiving feedback<br>Providing feedback	MCQ
10.	Which educational methods do you apply to tech NTS? More options are available.	Talking about NTS on a lecture<br>Talking about NTS on a practice<br>Praciticing NTS on bed-side sessions<br>Practicing NTS on simulation sessions<br>There is a whole session dedicated to NTS	MCQ
11.	How often do you teach NTS during a course?	Only on one session<br>On more sessions, however, not regularly<br>Regularly	MCQ

12.	Which of the followings are the most relevant to your method teaching NTS? You can choose more options.	We show/practice emergency situations highlighting the importance of teamwork.	MCQ
		We show/pracitce non-emergency situations highlighting the importance of cooperation with colleagues.	
		We show/pracitce non-emergency situations highlighting the importance of communication with patients.	
		We show/pracitce non-emergency situations highlighting the importance of communication with relatives.	
		We show/pracitce emergency situations highlighting the importance of proper decision making.	
		We show/pracitce non-emergency situations highlighting the importance of proper decision making.	
13.	The development of NTS education is required in our institute.		Likert-scale
		1: strongly disagree<br>6: strongly agree	
14.	Is teaching NTS in undergraduate education important?	Yes, because these skills are important for health-care workers.	MCQ
		Yes, because this is the trend today.	

		Yes, because appropriate NTS can improve patients' outcome.	
		No, it is not the role of undergraduate education.	
	Next questions should be applied if you do not teach NTS.		
15.	Why does your institute not teach NTS? Please choose the most relevant answer.	We think that teaching NTS is not important, because they do not influence the outcome of patietns' care.	MCQ
		We think that teaching NTS is not important, because the students will lern these skills during they work, after graduation.	
		The undergraduate education is not the place for teaching NTS.	
		We think that teaching NTS is improtant, but our curriculum does not count with teaching them.	
		We think that teaching NTS is improtant, but we do not have satisfactory technical support to teach them.	
		We think that teaching NTS is improtant, but we do not have satisfactory human resources to teaxh them.	
16.	Do you plan to introduce teaching NTS in the future?	Yes	MCQ

		No	
17.	Which methods could incorporated to your curriculum?	Talking about NTS on a lecture<br>Talking about NTS on a practice<br>Praciticing NTS on bed-side sessions<br>Practicing NTS on simulation sessions<br>Dedicating a whole session to NTS	MCQ
18.	Which NTS could be taught in your courses?	Communication with patients<br>Communication with colleagues<br>Teamwork<br>Situational awerness<br>Decision making<br>Adaptation<br>Receiving feedback<br>Providing feedback	MCQ
19.	Which of the following situations could be incorporated to ypur courses?	To show/practice emergency situations highlighting the importance of teamwork.<br>To show/pracitce non-emergency situations highlighting the importance of cooperation with colleagues.<br>To show/pracitce non-emergency situations highlighting the importance of communication with patients.	MCQ

	To show/pracitce non-emergency situations highlighting the importance of communication with relatives.
	To show/pracitce emergency situations highlighting the importance of proper decision making.
	To show/pracitce non-emergency situations highlighting the importance of proper decision making.

MCQ: multiple choice question
